# Chikungunya Virus Shedding in Semen: A Case Series

**DOI:** 10.3390/v14091879

**Published:** 2022-08-26

**Authors:** Ezequias B. Martins, Fernanda de Bruycker-Nogueira, Cintia D. S. Rodrigues, Carolina C. Santos, Simone A. Sampaio, Allison A. Fabri, Vinícius Guerra-Campos, Maria Angélica M. Mares-Guia, Nieli R. C. Faria, Aline S. Santos, Marcelle A. S. Pinto, Michele F. B. Silva, Isabella C. V. Moraes, Anielle Pina-Costa, Ana Maria B. Filippis, Patrícia Brasil, Guilherme A. Calvet

**Affiliations:** 1 Acute Febrile Illnesses Laboratory, Evandro Chagas National Institute of Infectious Diseases, Oswaldo Cruz Foundation, Rio de Janeiro 21045-900, Brazil; 2 Flavivirus Laboratory, Oswaldo Cruz Institute, Oswaldo Cruz Foundation, Rio de Janeiro 21045-900, Brazil

**Keywords:** chikungunya, semen, detection, genitals, shedding, persistence

## Abstract

Background: Chikungunya is a viral disease that is transmitted by mosquitoes. It is characterized by an acute onset of fever and severe arthralgia. Methods: We describe six cases of acute and post-acute chikungunya in which viral RNA was detected in semen. Conclusions: The most prolonged detection period was 56 days after illness onset. We attempted to cultivate positive semen samples, but virus isolation was unsuccessful in all cases.

## 1. Introduction

Chikungunya is a neglected tropical disease caused by chikungunya virus (CHIKV), an RNA arbovirus belonging to the Togaviridae family (genus, *Alphavirus*). CHIKV is transmitted by *Aedes aegypti* and *Aedes albopictus* mosquitoes [1]. Prior to 2013, CHIKV outbreaks were identified in Africa, Asia, Europe, and the Indian and Pacific Oceans. After 2013, the virus spread throughout most Americas, and arrived in Brazil in 2014 [2,3,4]. Chikungunya is characterized by intense joint pain with an abrupt onset, a high fever, and rash. The post-acute phase can involve recurrent joint pain and debilitating arthritis that may last for months or even years [5].

The detection and prolonged persistence of Zika virus (ZIKV) RNA in semen have been described in some studies [6,7]. In addition, prolonged persistence of Ebola and Marburg viruses in semen has been reported [8]. More recently, yellow fever virus and Chapare virus have been isolated from semen during the convalescent period of infection [9,10]. Although studies involving the isolation of CHIKV in semen samples are scarce, Bandeira et al. [11] reported CHIKV RNA in semen and urine samples 30 days after symptom onset. 

The knowledge of viral persistence in genital fluids is imperative to elucidate different forms of infection and possible reactional activity in response to the continuity of the virus in the body, which has relevance for the diagnosis and pathophysiology of viral diseases. Confirmation of the detection and persistence of the virus in semen is useful in health programs, especially for viruses with high rates of mortality or morbidity [12].

The present study aimed to describe the detection and duration of CHIKV RNA detected in semen samples obtained from six symptomatic men.

## 2. Materials and Methods

From 16 April 2019 to 10 October 2019, six male patients infected by CHIKV, and with the virus detectable in semen samples using real-time reverse transcriptase polymerase chain reaction (rRT-PCR), were followed for 3 months at the Acute Febrile Illnesses outpatient clinic of Oswaldo Cruz Foundation in Rio de Janeiro, Brazil. The patients were enrolled in a cohort study to evaluate the presence and duration of CHIKV infection in bodily fluid samples obtained from adult patients. The local ethics committee reviewed and approved the study (CAAE:06779019.0.0000.5262). Clinical data and samples (blood, urine, saliva, and semen) were collected at enrollment and every 15 days for 2 months, with a final collection at 3 months, for a total of six visits [13].

The samples were tested for CHIKV, ZIKV, and dengue virus (DENV) using rRT-PCR. RNA was extracted using the QIAmp Viral RNA Mini Kit. The general procedures for rRT-PCR for chikungunya, Zika, and dengue have been described elsewhere [14,15,16]. The rRT-PCRs mix was prepared using the GoTaq Probe 1-Step RT-qPCR System and was run using the Applied Biosystems 7500 Real-Time PCR System. Cycle threshold (Ct) values <38 and sigmoid curves were considered positive. Additional blood samples were collected to assess each patient’s hematological and biochemical parameters at each scheduled visit.

### 2.1. Testing the Conditions for Virus Isolation

To establish the conditions for viral isolation in cell culture, three semen samples with Ct 32, and one with Ct 34, with sufficient sample volume for testing and subsequent extractions, were initially diluted (1:4) in culture medium 199 without fetal bovine serum (FBS). Then, 100 µL was inoculated in a monolayer of VERO cells (kidney epithelial cells of an African green monkey) and grown in 12.5 cm^2^ flasks under four conditions: 1. The diluted inoculum was filtered with 0.22 µm syringe filter and removed from washing with 199 medium without FBS after adsorption; 2. The diluted inoculum was filtered with a 0.22 µm syringe filter and maintained after adsorption; 3. The diluted unfiltered inoculum was removed by washing with 199 medium without FBS after adsorption; 4. The diluted unfiltered inoculum was maintained after adsorption. All inoculums were adsorbed to the cell monolayer for one hour at 37 °C/5% CO_2_. 

After incubation, 1.5 mL of culture medium 199 containing 2.5% FBS was added. Flasks containing only the cell monolayer without manipulating the medium exchange were used as negative controls during the inoculation process. All flasks were incubated at 37 °C/5% CO_2_ for up to 14 days and monitored daily for cytopathic effects using an inverted microscope. Aliquots of 140 μL of supernatants from all flasks were taken at 3, 7, and 14 days of incubation, and were subjected to viral RNA extraction using the QIAamp Viral RNA Mini Kit (Qiagen, Inc., Hilden, Germany. https://www.qiagen.com/us/ accessed on 10 July 2020), according to the protocol described by the manufacturer, and stored at −70 °C. RNAs was tested for the detection of CHIKV by rRT-PCR, as described previously [14]. 

### 2.2. Virus Isolation

After testing the viral isolation conditions, all semen samples that were previously positive by rRT-PCR were inoculated in cell culture. The eight samples were diluted in culture medium 199 without FBS, six at a ratio of 1:2 (50 μL of semen + 50 μL of culture medium), and two samples at 1:10 (10 μL of semen + 90 μL of culture medium), owing to the scarcity of the original sample. A volume of 100 μL was inoculated into a monolayer of VERO cells grown in 12.5 cm^2^ flasks. The inoculums were not filtered and were maintained after adsorption. All inoculums were adsorbed to the cell monolayer for one hour at 37 °C/5% CO_2_, and, after incubation, 1.5 mL of culture medium 199 containing 2.5% FBS was added. Flasks were incubated at 37 °C/5% CO_2_ for up to 14 days and monitored daily for cytopathic effects using an inverted microscope. Aliquots of 140 μL of supernatant from all flasks were taken at 3, 7, and 14 days of incubation and were subjected to viral RNA extraction using the QIAamp Viral RNA Mini Kit (Qiagen, Inc., https://www.qiagen.com/us/ accessed on 10 July 2020), according to the protocol described by the manufacturer, and stored at −70 °C. RNA was tested for the detection of CHIKV by rRT-PCR, as described previously [14].

## 3. Results

All patients presented with symptomatic disease and moderate clinical manifestations. Their median age was 43.5 years (ranging, 33–56 years). There were no significant changes in the hematological or biochemical parameters at any of the study visits. All patients had chikungunya confirmed by detectable rRT-PCR in the serum during enrollment or seroconversion of anti-CHIKV-IgM. 

**Patient 1**: A 56-year-old male with hypertension and asthma complained of high fever (>40 °C), myalgia, asthenia, taste alteration, and moderate polyarthralgia for 4 days. He also described episodes of vomiting and nausea for 2 days. He developed a cutaneous rash 5 days after symptom onset. After the acute phase, mild symmetrical arthralgia in the shoulders, knees, and ankles resolved within six weeks without anti-inflammatory drug use.

**Patient 2:** A 48-year-old male previously healthy, with a 3-day history of fever, chills, headache, severe arthralgia, and prostration. On the seventh day, he developed a diffuse rash on the trunk, abdomen, and limbs. Joint pain was symmetrical (mainly in the shoulders, wrists, knees, and ankles) and lasted for more than six weeks. The patient was treated with prednisone (20 mg/day for 7 days) with a favorable outcome. Coinfection with DENV type 2 was detected.

**Patient 3**: A 51-year-old male with hypertension presented with a 4-day history of acute fever, malaise, generalized rash, and pain in multiple joints. The patient presented with edema in the hands and knees. The polyarthralgia was severe, lasted throughout the study period (three months), and was localized mainly in the knees. He was administered prednisone (20 mg/day for 14 days) and gabapentin (300 mg/day for 30 days) with partial improvement of his arthralgia.

**Patient 4**: A 33-year-old previously healthy man developed an abrupt onset of fever, chills, prostration, and moderate polyarthralgia affecting the shoulders, wrists, proximal interphalangeal joints, knees, ankles, and metatarsophalangeal joints. He had a 3-day history of a disseminated maculopapular rash. The fever lasted for 3 days, and mild joint pain lasted 8 weeks, without any pharmacological intervention. 

**Patient 5**: A 39-year-old man with three days of fever, chills, and sweating associated with severe pain in the shoulders, wrists, knees, ankles, and feet. Exanthema presented for 3 days. Polyarthralgia was severe and persisted throughout the follow-up period. Prednisone (20 mg/day for 20 days) was prescribed for partial pain relief. 

**Patient 6**: A 35-year-old man with a 5-day history of moderate fever, headache, prostration, myalgia, joint pain, and exanthema. The polyarthralgia was severe and lasted for more than 8 weeks. The patient received prednisone (20 mg/day for 21 days) with partial clinical relief of his symptoms. 

**Figure 1:**
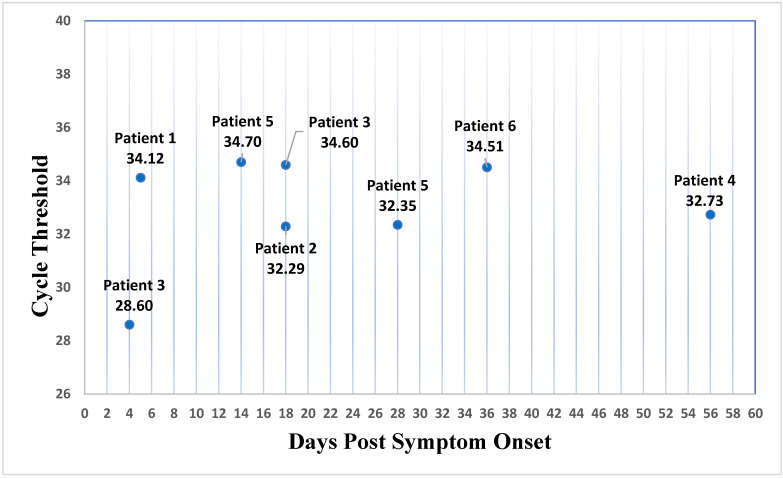
CHIKV cycle threshold by days after the onset of symptoms in semen.

Twenty-three semen samples were collected from these six patients, and eight were CHIKV-rRT-PCR-positive (Table 1). The maximum detection of CHIKV RNA in semen was 5, 18, 18, 56, 28, and 36 days after the onset of symptoms in patients 1 to 6, respectively (Figure 1). 

**Virus Isolation**

From the initial experiment to test the best conditions for viral inoculation, it was possible to recognize that there was no need for the filtering step of the semen samples to minimize possible bacterial contamination, since the cell cultures did not present typical turbidity, maintaining the transparent and translucent supernatant throughout the incubation period. After 3 days of inoculation, one of the four samples showed the beginning of morphological changes in the cell monolayer, resembling the expected cytopathic effect, progressing gradually over the next few days, with cell detachment after 7 days of inoculation. Such evolution initially occurred in cultures with diluted inoculums with and without filtration, and was maintained after adsorption. The same was observed later in the flasks that received the diluted inoculum of the same sample, with and without filtration, which was washed after adsorption. However, the rRT-PCR results of the supernatants from all samples collected at three points after incubation were negative. rRT-PCR of the original sample corresponding to the inoculations that would seem to progress in cytopathic effect was performed, but the result was negative.

A new viral inoculation was carried out with eight semen samples available under the conditions described in the methodology section. Three days after inoculation, two flasks showed complete detachment of the cell monolayer, one of which was inoculated with the sample diluted 1:10. Other cell monolayers began to show morphological changes, with cell detachment beginning 7 days after inoculation, and total detachment after 14 days. rRT-PCR of supernatants collected throughout the incubation period showed amplification with Ct 35 at the three collection points from a single sample initially diagnosed with Ct 28, which was considered as negative isolation, since the highest Ct maintained throughout the incubation period possibly refers to the inoculum remnant. All supernatants from the other samples tested negative.

## 4. Discussion

In this case series, CHIKV could be detected in semen samples from patients with either acute or convalescent disease. The longest CHIKV RNA detection period was 56 days after symptom onset in a 33-year-old, previously healthy man. All virus isolation attempts were unsuccessful for all samples.

Efforts to demonstrate virus detection in genital secretions of individuals infected with viruses that had previously been unknown to be sexually transmitted are necessary to investigate additional forms of transmission that could be of public health importance. Sexual transmission of Ebola virus was confirmed in Liberia in 2015 [17]. Sexual transmission of ZIKV has been reported for symptomatic male partners [18], and some studies have documented the prolonged detection of ZIKV RNA in semen [6,19]. Lalle et al. [20] described the presence of DENV RNA in semen up to 37 days post-symptom onset, when viremia and viruria were undetectable [20].

Bandeira et al. [11] described CHIKV RNA in semen and urine samples 30 days after symptom onset, and argued that prednisolone may have contributed to prolonged viral shedding. In our study, four patients were treated with prednisone; however, in this series, the patient with the highest viral detection in the semen (56 days) had not taken anti-inflammatory drugs.

Although CHIKV RNA was detected in the semen in our case series, the virus was not detected by rRT-PCR in the cultured samples. One explanation is that the cycle threshold values found in our samples were high, ranging from 28 to 34, which may correspond to a low viral load in semen. Viral cultures perform better with low Ct values in inoculated samples [18]. For example, low Ct values (20 to 28), viable infectious viruses, and prolonged shedding in semen have been reported in ZIKV studies and are related to sexual transmission [21,22]. Difficulty in viral isolation from the semen of patients, mainly with long-term viral shedding, has also been reported in studies of patients with ZIKV [23]. 

Factors intrinsic to the nature of the biological sample can influence the degradation of nucleic acid viruses by the presence of nucleases and the integrity of the viral particle, which can suppress viral infection and produce cytotoxic substances for cell culture [24,25]. In addition, storage of fresh samples without preservatives, as well as freezing and thawing processes, can be unfavorable for viral conservation, as demonstrated by the loss of detection of the viral genome in the original samples after re-extraction and repetition of rRT-PCR [26,27].

Additional limitations of our study were the small sample size and the short follow-up period. Infectivity is a prerequisite for pathogen transmission, and depends on factors such as the infectious dose and exposure route. Therefore, virus isolation remains the only direct and definitive approach for proving infectivity [28]. Twenty-seven different viruses have shown varying persistence in human semen [12]. The presence of viruses in semen may be more common than is currently understood, and viruses known as “non-sexually transmitted” should not be considered to be absent from genital secretions. All followed-up patients were informed about the possibility of being a carrier of CHIKV for a longer period, although we did not have confirmation of the risk of infectivity.

Studies on viral detection and semen persistence benefit clinical practice and public health, especially for viruses that can cause high chronic morbidity, such as CHIKV. Further studies are needed to evaluate the potential infectivity of semen and the sexual transmission of chikungunya.

## Figures and Tables

**Table 1:** Detection of CHIKV RNA in bodily fluids.

	Fluid	Visit 1	Visit 2	Visit 3	Visit 4	Visit 5	Visit 6
		Status (Ct)	Status (Ct)	Status (Ct)	Status (Ct)	Status (Ct)	Status (Ct)
**Patient 1**	**Semen**	**Positive (34.12)**	NC	Negative	Negative	Negative	NC
	**Serum**	**Positive (22.23)**	Negative	Negative	Negative	Negative	Negative
	**Urine**	Negative	Negative	Negative	Negative	Negative	Negative
	**Saliva**	**Positive (32.74)**	Negative	Negative	Negative	Negative	Negative
**Patient 2**	**Semen**	NC	**Positive (32.29)**	Missed	Negative	Missed	Missed
	**Serum**	**Positive (13.50)**	Negative	Missed	Negative	Missed	Missed
	**Urine**	Negative	Negative	Missed	Negative	Missed	Missed
	**Saliva**	**Positive (26.74)**	Negative	Missed	Negative	Missed	Missed
**Patient 3**	**Semen**	**Positive (28.06)**	**Positive (34.60)**	Negative	NC	NC	Missed
	**Serum**	**Positive (25.45)**	Negative	Negative	Negative	Negative	Missed
	**Urine**	Negative	Negative	Negative	Negative	Negative	Missed
	**Saliva**	**Positive (32.88)**	Negative	Negative	Negative	Negative	Missed
**Patient 4**	**Semen**	NC	Negative	Negative	**Positive (32.73)**	Negative	Negative
	**Serum**	Negative	Negative	Negative	Negative	Negative	Negative
	**Urine**	Negative	Negative	Negative	**Positive (35.49)**	Negative	Negative
	**Saliva**	Negative	Negative	Negative	Negative	Negative	Negative
**Patient 5**	**Semen**	NC	**Positive (34.70)**	**Positive (32.35)**	Negative	Negative	Missed
	**Serum**	**Positive (27.96)**	Negative	Negative	Negative	Negative	Missed
	**Urine**	Negative	Negative	Negative	Negative	Negative	Missed
	**Saliva**	Negative	Negative	Negative	Negative	Negative	Missed
**Patient 6**	**Semen**	Negative	Negative	**Positive (34.51)**	Negative	Negative	Missed
	**Serum**	**Positive (33.36)**	Negative	**Positive (36.65)**	Negative	Negative	Missed
	**Urine**	Negative	Negative	Negative	Negative	Negative	Missed
	**Saliva**	Negative	Negative	Negative	Negative	Negative	Missed

Ct: Cycle Threshold; NC: Not Collected; Missed: missed visit

## Data Availability

The data presented in this study are not publicly available but are available on request from the corresponding author.
